# The Depreciation of Medicine: Its Causes and Remedies

**Published:** 1906-10-06

**Authors:** 


### Editor's Letter-Box.

[Our Correspondents are reminded that prolixity is a great bar to publication, and that brevity of style and conciseness of statement greatly facilitate early insertion.]

### THE DEPRECIATION OF MEDICINE: ITS CAUSES AND REMEDIES.

*To the Editor of* The Hospital.

Sir,—I have read with interest your leading article on this subject. I think some of the causes lie in the natural difficulties which arise in every profession at the outset of a career, especially if the battle is complicated by

"Those twin gaolers of the daring heart
Low birth and iron fortune."

Many of our greatest consultants have known the uphill struggle against adversity which all have to undertake who desire to pay court to the most jealous of all goddesses—Medicina. Jenner, when over forty-five, lived in a small back room; Paget, if he had died at fifty, would have been a poor man; and many of the great ones now living (whose names it were invidious to mention) have considered themselves in luck's way when they dined upon a mutton chop once in a week. I have known practitioners rising to high rank in the profession who, at the outset of their career, have been seen at a fish-stall in the evening furtively purchasing the only meal of the day. All honour to these great ones.

It is a common public error to suppose that any man of education, and even good family, left to his own resources could at once earn from £300 to £600 a year. He would be lucky if he earned his living and kept the bailiffs from the door.

Your leading article mentions some of the causes which create this condition of affairs. Among others there is a systematic depreciation of medicine and an active propagation of falsehood by preachers who attribute epidemics to supernatural causes, by editors of religious or other inferior papers who are always ready to admit "matter" of this kind or any kind which will belittle medical authority. The wealthiest country in the world derives an ignoble revenue from casting the ægis of its protection over the rubbish advertised and sold by false and dishonest pretenders of knowledge. The patent medicine traffic amounts to nearly forty millions of packages, equivalent to nearly three millions of pounds sterling. This is the crowning proof of human imbecility which every self-respecting man or woman should expose and oppose.

Another cause is to be found in the monstrous philanthropy which is overshadowing the country and making beggary and pauperism respectable in general and special hospitals alike. I refer to the bloated out-patient departments of these institutions.

One other cause of the depreciation of medical services, however, has already been pointed out—the advance of new knowledge, which, unless the practitioner is more than usually alert, will leave him in the lurch—the advance in scientific disinfectants. The older school of practitioner belittled their use or used them cautiously, forgetting that a small band of expert chemists were making a study of bacteriology, following out the work of Pasteur and Lister. The handwriting was on the wall years ago for the practitioner to read. As has been already pointed out by a writer in the lay press, the new method must exercise an adverse influence on the earnings of the practitioner of the future.

In this country the medical profession seems to be either afraid or ashamed to assert itself. It need not be so, for it is a profession which possesses abundance of men of great adaptability and masters of many subjects. What is wanted is a better public opinion, and in order to obtain this the public must be enlightened and invited to listen to speeches of general interest connected with the diffusion and control of disease. The medical profession has this duty entirely in its own hands, and its members by a judicious use of their own powers could, in the course of a few years, elevate the now neglected and condemned medical knowledge to its proper place and set the public mind in a proper attitude towards the practitioners of the Divine Art of Healing. I am, etc.,

Iatros.

